# Construction of novel Bi_2_S_3_@Zn-Co-cLDHs heterojunction for enhanced photocatalytic degradation of levofloxacin with persulfate activation under visible light: mechanism and degradation pathway

**DOI:** 10.1039/d5ra03086b

**Published:** 2025-07-21

**Authors:** Nguyen Thi Mai Tho, Minh An Tran Nguyen

**Affiliations:** a Faculty of Chemical Engineering, Industrial University of Ho Chi Minh City Ho Chi Minh City Vietnam nguyenthimaitho@iuh.edu.vn

## Abstract

This study effectively synthesized the novel Bi_2_S_3_@Zn-Co calcined layered double hydroxides heterojunction (Bi_2_S_3_@ZC-cLDHs) *via* co-precipitation and thermal methods. ZC-LDHs built with a Zn^2+^/Co^2+^ molar ratio of 3 : 1, after calcination at 600 °C, yielded a blend of ZnO and ZnCo_2_O_4_ oxide, uniformly distributed on Bi_2_S_3_ rods. Bi_2_S_3_@ZC-cLDHs heterostructures exhibited superior photocatalytic efficiency for levofloxacin (LF) degradation compared to Bi_2_S_3_ and ZC-cLDHs under same catalytic conditions. The enhanced photodegradation efficiency results from the increased surface area and the establishment of a heterojunction at the interface of Bi_2_S_3_ rods and ZC-cLDHs. In addition, the photocatalytic degradation efficiency of LF enhanced from 74.8% to 90.1% with the addition of persulfate (PS) as an activating under visible light, utilizing a catalyst loading of Bi_2_S_3_@ZC-cLDHs at 1.0 g L^−1^, initial concentration of 20 ppm, PS loading of 0.25 g L^−1^, and light exposure duration of 90 minutes. The Z-scheme established the photocatalytic mechanism for the degradation of LF using Bi_2_S_3_@ZC-cLDHs with PS activation. Radical trapping tests demonstrated that O_2_˙^−^ and h^+^ were the significant active species. The combination of PS and catalyst had a synergistic effect, wherein S_2_O_8_^2−^ interacted with electrons to create SO_4_˙^−^ during the photocatalytic process. The analysis using LC-MS provided a thorough understanding of possible photocatalytic breakdown path of LF; the photoproducts were small-sized molecules with little impact on the environment.

## Abstract 2

This study effectively synthesized the novel Bi_2_S_3_@Zn-Co calcined layered double hydroxides heterojunction (Bi_2_S_3_@ZC-cLDHs) *via* co-precipitation and thermal methods.

## Graphical abstract

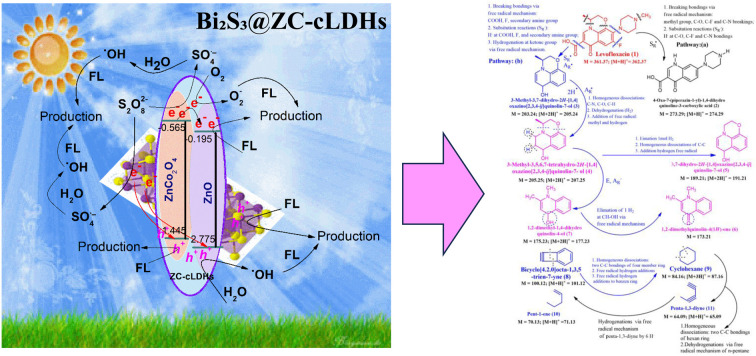

## 1 Introduction

Antibiotics are pharmacological agents that may suppress bacterial proliferation, therefore used for the treatment of bacterial infections, inflammation, and several other diseases.^1,2^ Levofloxacin (LF) is a third-generation fluoroquinolone antibiotic that has a wide range of antibacterial action in both people and animals.^3^ LF has significant chemical stability, bio-refractory properties, demonstrates little metabolic clearance in humans, and is released into the environment post-usage.^4,5^ The discharged LF may ultimately enter water sources, leading to the emergence of drug-resistant bacteria and antibiotic resistance, posing a threat to aquatic creatures and human health.^6^ Consequently, finding reliable and practical ways to eliminate medicines like LF from the aquatic environment is thus urgent.

Layered double hydroxides (LDHs) are anionic clay minerals characterized by a layered structure and the general formula [M^2+^_1−*a*_M^3+^_*a*_(OH)_2_]^*a*+^·[A^*n*−^_*a*/*n*_]·*m*H_2_O, where M^2+^ represents a divalent metal cation and M^3+^ denotes a trivalent metal cation, has a positively charged octahedral structure.^7,8^ It equilibrates these positively charged layers by interposing negatively charged inorganic or organic anions between them. LDHs serve as precursors for cLDHs upon calcination at an appropriate temperature, resulting in the decomposition of LDHs into cLDHs, which include divalent metal oxides and spinel oxides. The capacity of cLDHs to restructure LDHs *via* the method of “memory effect” has led to prompted significant research in the domain of adsorption.^9,10^

Nowadays, advanced oxidation processes (AOPs) using semiconductor photocatalysis have become a crucial and highly endorsed method for the effective degradation of organic pollutants, especially antibiotics, due to their high efficiency, cost-effectiveness, simplicity, and energy conservation.^1,11,12^ Numerous semiconductor materials have been investigated potentially photocatalytic materials by absorbing visible light and producing reactive free radicals (ROS) ˙OH, O_2_˙^−^; photogenerated electrons, and holes (e^−^/h^+^) for the degradation of organic waste.^13,14^ The special interactive effects of the two oxide components of cLDHs with larger surface areas than LDHs have led to the recent widespread application of cLDHs in photocatalysis.

Certain research has shown considerable success in using cLDHs or cLDHs heterostructures as photocatalysts for the degradation of contaminants such as CoFe-cLDHs,^7^ NiCoFe-LDHs, Ti-MOF/NiFeLDHs^8^ nevertheless, there is a limitation of studies on ZnCo-cLDHs.

Wide light absorption range, effective charge separation, superior redox potential, and exceptional stability are all desirable properties of a photocatalyst.^3^ Upon evaluating the characteristics, pure semiconductors exhibit notable deficiencies, including insufficient band gap energy in Bi_2_S_3_, resulting in rapid recombination of e^−^/h^+^ pairs,^15,16^ and excessive band gap energy in ZnO,^17^ which hinders light absorption, thereby contributing to the low efficiency of organic matter decomposition. To increase the durability of materials and the efficiency of photoinduced pair separation, researchers have implemented several active strategies, which includes doping metals and decorating substrate on semiconductors, or coupling with other semiconductors to create heterojunction photocatalysts.^3,14,18^

In addition, to improve the catalytic efficacy of semiconductors, an enhanced oxidation technique using activated persulfate has been investigated for pollutant treatment.^19,20^ Usually, PS does not directly interact with pollutants; however, it is activated by solar-driven photocatalytic materials to generate reactive species, hence improving its capacity to decompose pollutants. At this time, persulfate and catalyst have a synergistic effect; S_2_O_8_^2−^ quickly traps photogenerated electrons to produce SO_4_˙^−^, decreasing the recombination of charge carriers, and SO_4_˙^−^ may also interact with H_2_O to provide ˙OH radicals, therefore augmenting the capacity to destroy organic substances.^7,21,22^

From the researched strategies to improve the photocatalytic efficiency of semiconductor materials, specifically cLDHs. In this work, we addressed the following problems: (1) synthesis of cLDHs materials using LDHs precursors obtained from cobalt and zinc salts *via* the co-precipitation method, disperse on the surface of Bi_2_S_3_ rods (Bi_2_S_3_@ZC-cLDHs). (2) A comprehensive examination of the photocatalytic of Bi_2_S_3_@ZC-cLDHs for activating PS to degrade LF antibiotics in simulated light conditions. (3) Investigation of the photocatalytic mechanism of Bi_2_S_3_@ZC-cLDHs, activation mechanism of PS, and identification of possible degradation pathway of LF. (4) Evaluating the reusability and stability of Bi_2_S_3_@ZC-cLDHs heterostructures.

## 2 Experimental

### 2.1 Materials and chemicals

Bismuth(iii) nitrate pentahydrate (Bi(NO_3_)_3_·5H_2_O, 98.0%), cobalt(ii) nitrate hexahydrate (Co(NO_3_)_2_·6H_2_O; 98%); zinc acetate dihydrate (Zn(CH_3_COO)_2_·6H_2_O, 98%), sodium hydroxide (NaOH, 99%) was purchased from Sigma-Aldrich, USA. Ethylene glycol ((CH_2_OH)_2_, 99%), levofloxacin ((C_18_H_20_FN_3_O_4_) 99%); ethanol (C_2_H_5_OH, 99.5%); *tert*-butanol (99.5%); *p-*benzoquinone; ethylenediamine tetra acetic acid disodium salt (Na_2_EDTA, 99%); cexadecyl trimethyl ammonium bromide (CTAB, 99%) and thiourea (H_2_NCSNH_2_, 99%), were purchased from Xilong Scientific Co., Ltd China.

### 2.2 Synthesis of Bi_2_S_3_@ZC-cLDHs

#### 2.2.1 Synthesis of Bi_2_S_3_

At a rate of 2 mL min^−1^, the precursor solution of 1.91 g Bi(NO_3_)_3_·5H_2_O dissolved in 50 mL ethylene glycol is gradually added to the solution of 0.46 g thiourea and 0.05 g CTAB dissolved in 50 mL ethylene glycol (the molar ratio of Bi^3+^/S^2−^ is 2/3). The reaction occurs at room temperature in an ultrasonic bath. The resulting precipitate mixture is placed in a round-bottom flask, afterwards heated and stirred continuously at 170 °C for 14 hours. The precipitate is filtered, rinsed with deionized water and pure alcohol, and then dried at 100 °C to provide a fine black powder (Bi_2_S_3_).

#### 2.2.2 Synthesis of Bi_2_S_3_@ZC-cLDHs

Bi_2_S_3_@ZC-cLDHs, mass ratio of Bi_2_S_3_/ZC-cLDHs of 15%, was synthesized using the co-precipitation and thermal method. Disperse 0.5 g of Bi_2_S_3_ and 0.05 g of CTAB in 50 mL of NaOH 1 M solution using an ultrasonic bath for 30 minutes to create a suspension. Add 100 mL of Zn(CH_3_COO)_2_ 0.3 M and Co(NO_3_)_2_ 0.1 M solution into the previous suspension at a rate of 2 mL min^−1^, maintain the pH 10 using 1 M NaOH solution and ensure continuous stirring during the process. The precipitate mixture was aged for 8 hours at 100 °C. The precipitate was thereafter filtered and repeatedly washed with deionized water until the washing water of the precipitate reached pH 7. The precipitate was then cleaned with ethanol and dried at 100 °C to produce a black solid known as Bi_2_S_3_@ZC-LDHs. Bi_2_S_3_@ZC-LDHs were calcined for 4 hours at 600 °C at a rate of 5 °C min^−1^ to produce Bi_2_S_3_@ZC-cLDHs. The ZC-cLDHs sample was synthesized in a manner like Bi_2_S_3_@ZC-cLDHs, except the starting solution included just 50 mL of NaOH 1 M and 0.05 g of CTAB, skipping 0.5 g of Bi_2_S_3_.

### 2.3 Photocatalytic activity evaluation

The photocatalytic efficacy of the synthesized samples was evaluated for the degradation of LF using PS as an activator under visible light. The photocatalytic occurred in a catalytic system including 0.1 g of Bi_2_S_3_@ZC-cLDHs evenly spread in 100 mL of LF 20 ppm (*C*_in_). The photocatalytic system has two glass layers; the inner layer is LF solution and catalyst to create a uniformly stirred suspension throughout the reaction, while the outer layer features a steady water circulation system kept at the room temperature. A 300 W halogen lamp (64 640 HLX 150 W, 24 V, Osram, Germany) simulating visible light was emitted with a maximum intensity of approximately 100 mW cm^−2^, directed at the suspension. Before being exposed to lighting, the suspension is stirred in darkness for 30 minutes, for the suspension establishes equilibrium/desorption, following which the LF concentration (*C*_o_) is measured. The loading of PS activator is introduced into the photocatalytic system during the commencement of illumination, which lasts for 90 minutes. 5 mL of the suspension in the system are removed and filtered through a 0.22 mm filter to eliminate solids after fifteen minutes of illumination. With wavelength of 290 nm for LF, the UV-vis spectrophotometer was used to track the concentration of the degenerated solution (*C*_*t*_) over time. To examine the variables impacting the photocatalytic process, the influencing parameters will be altered while the other parameters stay unchanged. Analysis of reactive oxygen species, suitable quantities of *p*-benzoquinone (*p*-BQ), methanol (MeOH), *tert*-butanol (TBA), and Na_2_-EDTA were added into the photocatalytic system before beginning light irradiation.

## 3 Results and discussion

### 3.1 Characterization

**Fig. 1:**
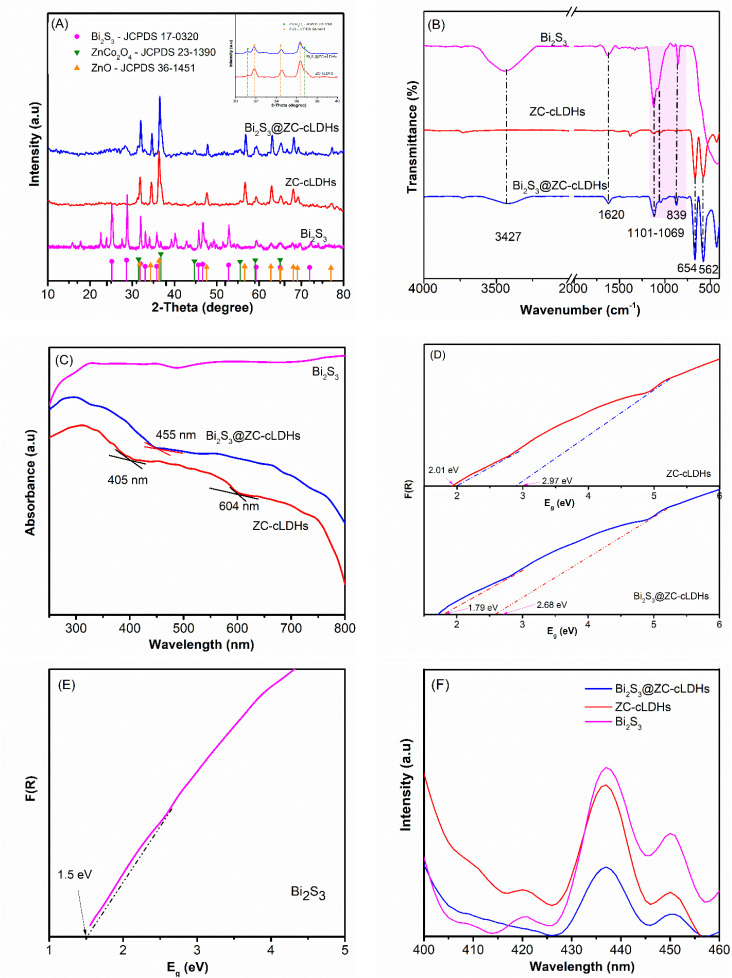
(A) XRD; (B) FT-IR; (C) UV-Vis-DRS ; (D and E) the *E*_g_ and (F) PL of Bi_2_S_3_, ZC-cLDHs, and Bi_2_S_3_@ZC-cLDHs samples.

The X-ray diffraction patterns (XRD) of the Bi_2_S_3_, ZC-cLDHs, and Bi_2_S_3_@ZC-cLDHs as synthesized samples are shown in Fig. 1A. The ZC-cLDHs sample exhibits peaks at 2*θ* positions 31.77°; 34.34°; 36.56°; 47.61°; 56,61°; 62.82°; 65.13°; 68.12° and 69.11° corresponding to the (100), (002), (101), (102), (110), (200), (112), and (004) planes, aligning with the peaks of ZnO^23^ (JCPDS, 36-1451), and the peaks at 2*θ* positions 31.5°; 36.76°; 44.70°; 55.52°; 59.11° and 65.01° correspond to the (220), (311), (400), (422), (511) and (440) planes, recognizing with the standard peaks of ZnCo_2_O_4_ (refs. 24 and 25) (JCPDS-23-1390). Fig. 1A at 2*θ* from 30 to 40° shows the overlap of the diffraction peaks at locations 31.77° and 36.56° of ZnO and 31.5° and 36.76° of ZnCo_2_O_4_.

The peaks at 2*θ* positions 25.17°; 28.65°; 31.93°; 33.05°; 35.81°.45.55°; 46.67°; 52.79°; 59.33°. 65.02° and 71.98° of the Bi_2_S_3_ sample match with the typical peaks of Bi_2_S_3_ (ref. 26 and 27) (JCPDS No. 17-0320). The Bi_2_S_3_@ZC-cLDHs sample, with a mass ratio of Bi_2_S_3_/ZC-cLDHs at 15%, exhibited diffraction results indicating that, aside from the characteristic peaks of ZnO and ZnCo_2_O_4_ phase, the low-intensity peaks of Bi_2_S_3_ were either not distinctly visible or nearly covered up by the obvious peaks of ZnO and ZnCo_2_O_4_, with only the intensity of the strong peaks at the position of Bi_2_S_3_ being obvious. Furthermore, the peaks were distinct and acute, with no anomalous peaks present, indicating that the synthesized materials exhibited great purity and few contaminants.

Fig. 1B presents the Fourier transform infrared spectroscopy (FT-IR) of Bi_2_S_3_, ZC-cLDHs, and Bi_2_S_3_@ZC-cLDHs samples. The Bi_2_S_3_ sample exhibits two peaks at 1101–1069 cm^−1^ and 839 cm^−1^, corresponding to the vibrations of the Bi–S bond.^28,29^ The bands at 1620 cm^−1^ and 3347 cm^−1^ correspond to the bending and stretching vibrations of O–H in adsorbed H_2_O. The ZC-cLDHs exhibit two vibrational frequencies at 654 and 562 cm^−1^, corresponding to the Zn–O and Co–O bonds,^21,30^ respectively. Specifically for the Bi_2_S_3_@ZC-cLDHs sample, along the vibrations of Zn–O and Co–O bonds, Bi–S vibrations are also present, indicating an interaction between Bi_2_S_3_ and ZC-cLDHs that results in the establishment of Bi_2_S_3_@ZC-cLDHs heterostructures.

Diffuse reflectance spectroscopy (UV-Vis DRS) was used for determining the optical absorption of the synthesized samples.^30^ The findings demonstrate that the absorption spectrum of Bi_2_S_3_ extends from the visible light spectrum to the infrared region.^15^ ZC-cLDHs demonstrates two absorption wavelengths at 405 and 604 cm^−1^. The XRD findings indicate that ZC-cLDHs has two phases: ZnO and ZnCo_2_O_4_. Consequently, the absorption wavelength at 405 nm corresponds with ZnO,^31^ while the absorption wavelength at 604 nm belongs to ZnCo_2_O_4_.^32^ The combination of ZC-cLDHs with Bi_2_S_3_ results in Bi_2_S_3_@ZC-cLDHs exhibiting an absorption edge at 455 nm, which is blue-shifted relative to ZC-cLDHs. This shift may result from the interaction between Bi_2_S_3_ and ZC-cLDHs, which modifies the energy band, transitioning from visible light to infrared light. The results of determining the maximum absorption wavelength of the synthesized materials and calculating the band gap energy (*E*_g_) using the Kubelka–Munk function^33^ are shown in Fig. 1C. ZC-cLDHs has an absorption wavelength of 405 nm, corresponding to an energy gap (*E*_g_) of 2.01 eV for ZnCo_2_O_4_, and wavelength of 604 nm, corresponding to 2.97 eV for ZnO. Bi_2_S_3_@ZC-cLDHs is characterized by two *E*_g_ values of 1.79 eV and 2.68 eV, respectively (Fig. 1D). According to Fig. 1E the Bi_2_S_3_ sample is determined to have *E*_g_ = 1.5 eV.

The photoluminescence (PL) intensity correlates with the amplitude of the photogenerated electron–hole separation.^34^ The carrier separation increases as the PL intensity decreases, indicating enhanced photodegradation efficacy.^35^Fig. 1F illustrates the PL spectra of ZC-cLDHs; Bi_2_S_3_ and Bi_2_S_3_@ZC-cLDHs, all measured at an excitation wavelength of 320 nm. The image clearly indicates that ZC-cLDHs and Bi_2_S_3_ exhibit robust fluorescence, indicating rapid recombination of electrons and holes.^34^ The fluorescence intensity of the synthesized sample is much lower than that of Bi_2_S_3_@ZC-cLDHs, suggesting that the combination of Bi_2_S_3_-ZC-cLDHs efficiently suppresses electron–hole recombination, hence enhancing the LF breakdown of the composites.^36^ Bi_2_S_3_@ZC-cLDHs are expected to function well as photocatalysts within the visible light spectrum.

### 3.2 XPS

**Fig. 2:**
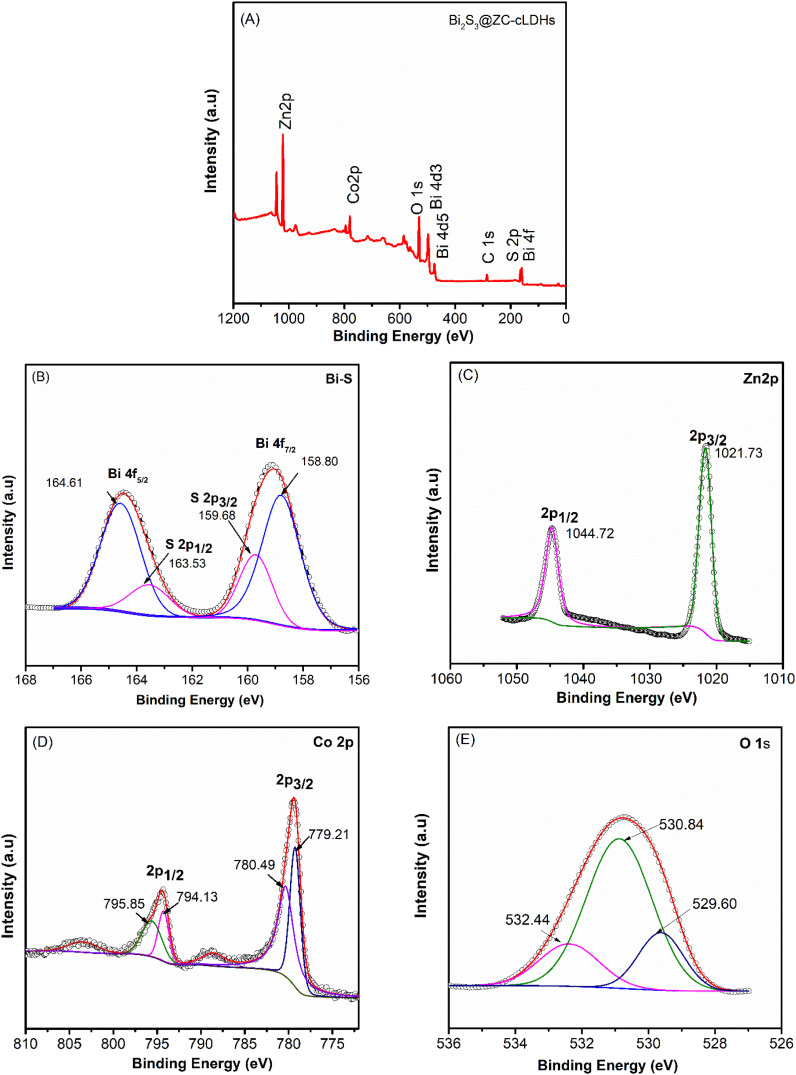
XPS survey spectra (A); high resolution core level spectra of (B) Bi–S; (C) Zn 2p; (D) Co 2p; and (E) O 1s for Bi_2_S_3_@ZC-cLDHs.

X-ray photoelectron spectroscopy (XPS) was used for evaluating the surface chemical composition and elemental valence state of the Bi_2_S_3_@ZC-cLDHs composite,^33^ as seen in Fig. 2. The full scan spectrum of Bi_2_S_3_@ZC-cLDHs reveals the presence of Zn, Co, Bi, and S with no other elements detected, indicating that the synthesized sample exhibits great purity (Fig. 2A). The peak of C 1s is applied to a reference for charge correction. The atomic percentages of C, O, Co, Zn, Bi and S are 24.08%; 47.74%; 10.29%; 16.48%; 0.75%; 0.66% respectively. Zn 2p_1/2_ and Zn 2p_3/2_ have binding energy peaks at 1044.72 eV and 1021.73 eV in the high-resolution spectrum of Zn (Fig. 2B). The high-resolution XPS spectra of Bi 4f and S 2p exhibits two significant symmetric peaks at binding energy levels of 164.61 eV and 158.8 eV, typical of Bi 4f_2/5_ and Bi 4f_7/2_, confirming that Bi is in the Bi^3+^ oxidation state.^15,37^ The peaks of Bi 4d_3/2_ and Bi 4d_5/2_, respectively, at 470 and 495 eV are also visible in Fig. 2A. The two energy peaks at 159.68 eV and 163.54 eV are situated between Bi 4f_5/2_ and Bi 4f_7/2_, indicative of S 2p_3/2_ and S 2p_1/2_.^16^

This result implies the establishment of Bi–S bonds in Bi_2_S_3_-ZC-cLDHs. Fig. 2C exhibits two binding energies at 795 eV and 780 eV, corresponding to Co 2p_1/2_ and Co 2p_3/2_, respectively.^25^ The deconvoluted results for these two binding energy peaks indicate that there are two energy peaks at 795.85 eV and 780.49 eV, typical of Co^2+^, and two energy peaks at 794.13 eV and 779.21 eV, corresponding to Co^3+^, in the spinel complex ZnCo_2_O_4_.^33,38^ Furthermore, two satellite peaks are seen at energy peaks of around 788 and 802 eV, indicating the existence of multivalent cobalt.^38^ The deconvolution at the 531 eV binding energy of O 1s shows three energy values: 529.6,530.84, and 532.44 eV (Fig. 2E). The binding energy of lattice oxygen (O_L_) at 529.6 eV corresponds to M–O bonds.^39^ The strong binding energy peak at 532.44 eV indicates a significant presence of oxygen vacancies (O_V_), and the energy position of 532.44 eV represents the adsorption of oxygen on the surface.^40,41^

#### 3.2.1 SEM and HrTEM

**Fig. 3:**
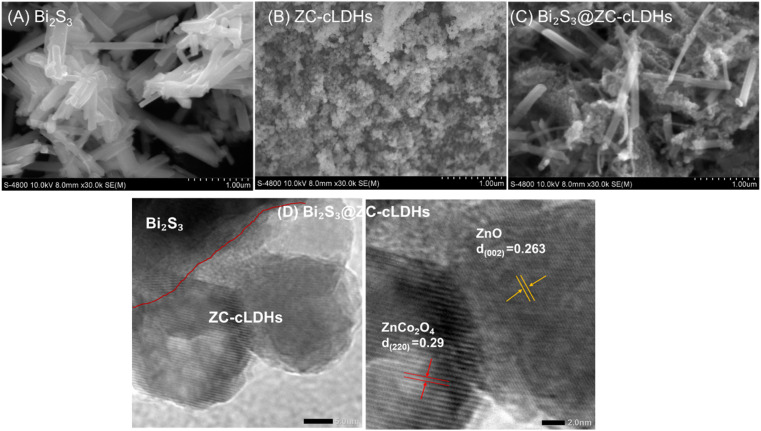
SEM of (A) Bi_2_S_3_, (B) ZC-cLDHs, (C) Bi_2_S_3_@ZC-cLDHs samples; TEM of (D) Bi_2_S_3_@ZC-cLDHs sample.

Fig. 3 displays the SEM image findings of the Bi_2_S_3_, ZC-cLDHs, Bi_2_S_3_@ZC-cLDHs samples. The Bi_2_S_3_ sample exhibits a rod-like morphology, with a diameter of 100 nm and an approximate length of 1 μm, organized in a disordered fashion. ZC-cLDHs exhibit both spherical and hexagonal shapes, with layers piled chaotically and unevenly because of layer structural collapse after heating at 600 °C. SEM images of Bi_2_S3@ZC-cLDHs show that ZC-cLDHs clusters adhere and disperse on the surface of Bi_2_S_3_ rods. This enlarges the contact surface, complicating their adherence during interaction with the solution. The distinctive architecture of the Bi_2_S_3_@ZC-cLDHs composites enhances the photocatalytic efficacy of this heterojunction. The HRTEM image (Fig. 3D) of Bi_2_S_3_@ZC-cLDHs distinctly delineates the boundaries of three phases, with the spherical morphology of cLDHs comprising ZnO and ZnCo_2_O_4_ distributed on the Bi_2_S_3_ rods, exhibiting lattice spacings of 0.263 nm and 0.29 nm corresponding to the (002) plane of ZnO and the (220) plane of ZnCo_2_O_4_, respectively.^41–44^

**Fig. 4:**
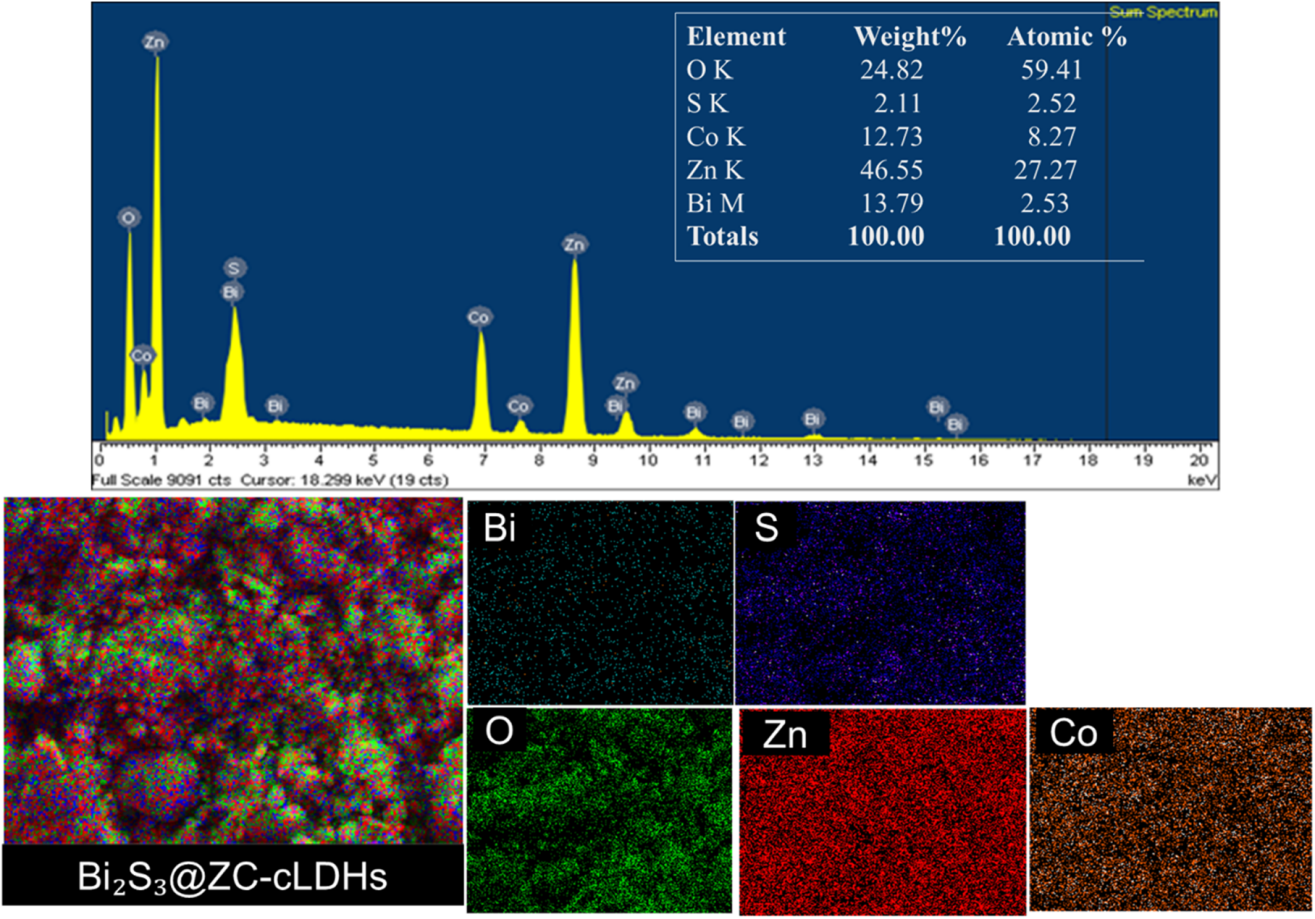
EDX, EDX elemental mapping image of Bi_2_S_3_@ZC-cLDHs.

The results of EDX analysis and EDX mapping for the Bi_2_S_3_@ZC-cLDHs sample are shown in Fig. 4. The EDX spectrum of the Bi_2_S_3_@ZC-cLDHs sample reveals the presence of elements Bi, S, Co, Zn, and O, with mass percentages of 13.79%, 2.11%, 12.73%, 46.55%, and 24.82%, and atomic percentages of 2.53%, 2.52%, 8.25%, 27.27%, and 59.41%, respectively. The EDX maping revealed the elemental composition of the Bi_2_S_3_@ZC-cLDHs, providing insight into the distribution of Bi, S, Co, Zn, and O within the composites.

### 3.3 Photocatalytic activity

#### 3.3.1 Effect of photocatalytic conditions

**Fig. 5:**
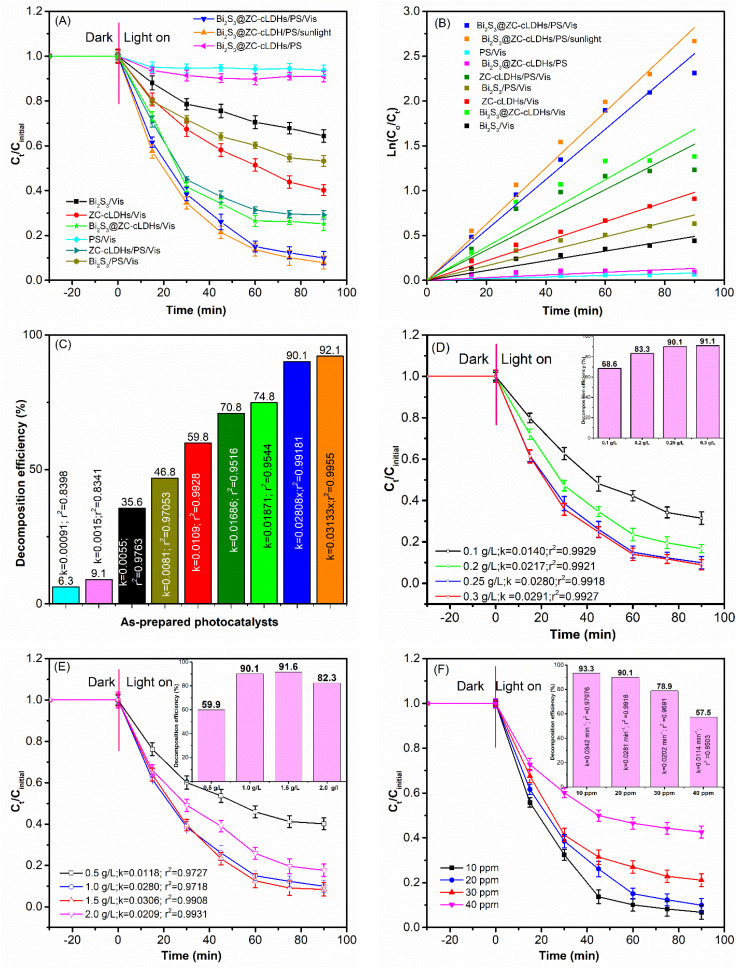
(A) Photocatalytic activity, (B) reaction kinetics; (C) photocatalytic efficiency of LF degradation on different conditions. Photocatalytic activity, reaction kinetics, photocatalytic efficiency of LF degradation on different (D) PS dose, (E) dosage of Bi_2_S_3_@ZC-cLDHs; (F) initial LF concentration.

Fig. 5A–C shows the photocatalytic activity efficiency of as-synthesized samples in the decomposition of LF under different conditions. The results indicated that the samples were unable to absorb LF antibiotics within 60 minutes but were able to have photocatalytic activity to break down LF after 90 min irradiation.

The research assessed the photocatalytic efficacy of Bi_2_S_3_, ZC-cLDHs, and Bi_2_S_3_@ZC-cLDHs samples under identical catalyst loading conditions of 1.0 g L^−1^ and LF concentration of 20 ppm in two scenarios: (i) simulated sunshine (Bi_2_S_3_/Vis; ZC-cLDHs/Vis; Bi_2_S_3_@ZC-cLDHs/Vis). (ii) Simulated sunshine in conjunction with a dose of 0.25 g L^−1^ PS activator (Bi_2_S_3_/PS/Vis; ZC-cLDHs/PS/Vis; and Bi_2_S_3_@ZC-cLDHs/PS/Vis).

The experimental findings demonstrated that the combination of Vis and PS significantly enhanced the LF degradation efficiency of Bi_2_S_3_, ZC-cLDHs, and Bi_2_S_3_@ZC-cLDHs, suggesting that simulated sunlight activates PS to promote LF degradation. The studies indicated that Bi_2_S_3_/Vis; ZC-cLDHs/Vis and Bi_2_S_3_@ZC-cLDHs/Vis exhibited a photocatalytic efficiency of 35.8% (*k* = 0.02055 min^−1^) 59.8% (*k* = 0.0109 min^−1^) and 74.8% (*k* = 0.01686 min^−1^), whereas Bi_2_S_3_/PS/Vis; ZC-cLDHs/PS/Vis and Bi_2_S_3_@ZC-cLDHs/PS/Vis had a photocatalytic efficiency of 46.7% (*k* = 0.00818 min^−1^); 74.8% (*k* = 0.01872 min^−1^). and 90.1% (*k* = 0.02808 min^−1^). The photocatalytic efficiency of LF degradation of Bi_2_S_3_; ZC-cLDHs and Bi_2_S_3_@ZC-cLDHs with PS activator is higher than that without activator in the visible light region. In which, the LF decomposition rate of the Bi_2_S_3_@ZC-cLDHs/PS/Vis increased by over two-fold compared to the Bi_2_S_3_@ZC-cLDHs/Vis, as S_2_O_8_^2−^ carried out the electron reaction, leading to the formation of SO_4_˙^−^. This then interacted with H_2_O to produce ˙OH, enhancing the LF decomposition,^45,46^ according to eqn (i) and (ii).

i S_2_O_8_^2−^ + e^−^ → SO_4_˙^−^ + SO_4_^2−^

ii SO_4_˙^−^ + H_2_O → SO_4_^2−^ + ˙OH + H^+^

The results indicate that the degrading efficiency of LF was not significantly diminished (6.3%) when the PS activator was paired with simulated irradiation light in the experiment. This suggests that the PS could only scarcely be activated to degrade LF in the absence of a catalyst. Furthermore, the findings of the experiment demonstrated that the photocatalytic efficacy of LF degradation of pure Bi_2_S_3_; ZC-cLDHs was rather low in both Vis and PS/Vis. A comparative investigation of the LF degradation efficiency of Bi_2_S_3_/PS/Vis and ZC-cLDHs/PS/Vis revealed that Bi_2_S_3_ and ZC-cLDHs had modest LF degradation efficiencies, ranging from around 46.8% to 70.8%. The findings indicated that the amalgamation of Bi_2_S_3_ and ZC-cLDHs significantly enhanced the photocatalytic efficiency for LF degradation to 90.1%. The photocatalyst performance was determined by the values of *k*, which were subsequently arranged in descending order of Bi_2_S_3_@ZC-cLDHs/PS/Vis (*k* = 0.02808 min^−1^) > ZC-cLDHs/PS/Vis (*k* = 0.01686 min^−1^) > Bi_2_S_3_/PS/Vis (0.0081 min^−1^). Similarly, the findings indicated that Bi_2_S_3_@ZC-cLDHs/Vis had a superior LF degradation efficiency of 74.5%, in contrast to Bi_2_S_3_/Vis (35.8%) and ZC-cLDHs/Vis (59.8%). The efficacy of the photocatalyst was assessed based on the *k* values, which were organised in decreasing order as follows: Bi_2_S_3_@ZC-cLDHs/Vis (*k* = 0.01871 min^−1^) > ZC-cLDHs/Vis (*k* = 0.0109 min^−1^) > Bi_2_S_3_/Vis (*k* = 0.0055 min^−1^).

The photocatalytic effects of Bi_2_S_3_@ZC-cLDHs heterojunction surpassed those of pure Bi_2_S_3_ and ZC-cLDHs, indicating that this heterojunction enhanced photocatalytic performance. The UV-Vis/DRS results show that Bi_2_S_3_ exhibits an absorption wavelength range from the ultraviolet to the visible the spectrum, with a low band gap energy. ZC-cLDHs comprises ZnCo_2_O_4_ with an *E*_g_ of 2.01 eV, and ZnO with an *E*_g_ of 2.97 eV. The Bi_2_S_3_@ZC-cLDHs sample exhibits a chemical interaction between ZC-cLDHs and Bi_2_S_3_ that alters the light absorption spectrum. The interaction between Bi_2_S_3_ and ZC-cLDHs form a heterojunction, leading to a synergistic impact between the two phases. This process, Bi_2_S_3_ establishes efficient intermediate energy levels that facilitate the transfer between electrons and holes, therefore decreasing the possibility of recombination between photogenerated e^−^/h^+^, enhancing the ability to decompose LF.^15,30,31^ Another characteristic is the material's morphology; the SEM maging results indicate that Bi_2_S_3_ has a rod-like structure, whereas ZC-cLDHs exhibits nanolayered structure. The precipitation of ZC-cLDHs on Bi_2_S_3_ enhances dispersion capability and surface area, reduces the aggregation tendency of Bi_2_S_3_-ZC-cLDHs, and facilitates light absorption, so expanding the catalytic efficacy of the composite.^15^

In addition, the photocatalytic activity of Bi_2_S_3_@ZC-cLDHs heterojunction for LF degradation was tested under natural light (Bi_2_S_3_@ZC-cLDHs/PS/sunlight); the experimental time in natural light was from 10 to 12 h local time. The degradation efficiency of Bi_2_S_3_@ZC-cLDHs/PS/sunlight was 92.1% greater than that of Bi_2_S_3_@ZC-cLDHs/PS/Vis, possibly due to the higher light intensity of sunlight compared to that provided by the Orsam lamp. Furthermore, Bi_2_S_3_@ZC-cLDHs have the capability to absorb both visible and ultraviolet spectra in natural light.^47,48^

The first-order kinetic equation, 
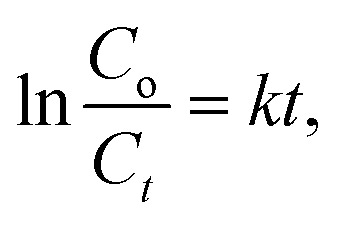
 was used to model the LF breakdown process of Bi_2_S_3_@ZC-cLDHs based on the experimental results. *C*_o_, *C*_*t*_ (mg L^−1^): initial concentration and the concentration of LF at any instant time *t*; and *k*: the rate constant (min^−1^).

Fig. 5B and C displays the linear equation, rate constant, and correlation coefficient *r*^2^ for the LF decomposition process of materials. The findings indicate that the *r*^2^ value varies from 0.9516–0.9918, demonstrating that the first-order apparent kinetic equation is entirely appropriate for assessing the LF conversion kinetics with the PS activator of the Bi_2_S_3_@ZC-cLDHs. The photocatalytic efficiency of the PS-activated Bi_2_S_3_@ZC-cLDHs sample is most effective, achieving 90.1%, with a rate constant of *k* = 0.02808 min^−1^. Table 1 displays comparisons between the Bi_2_S_3_@ZC-cLDHs synthesized in this study and other reported photcatalysts for the elimination LF with or without an activator PS. The Bi_2_S_3_@ZC-cLDHs compound catalyst with PS activator demonstrated a better LF degradation efficiency than the other catalysts.

**Table 1:** Levofloxacin degradation efficiency is exposed to visible light using various photocatalysts

Photocatalysts	Catalyst dose (g L^−1^)	Levofloxacin concentration (mg L^−1^)	Activation	Degradation efficiency	Ref.
Ag_3_PO_4_/C_3_N_4_/ZnO	0.5	100 mL; 10		89.2%	49
AgFeO_2_/Ag_3_VO_4_	0.2	50 mL; 20		95.95%	3
Co_3_O_4_/Bi_2_MoO_6_@g-C_3_N_4_	1.0	50 mL; 25		95.21%	36
Co-Bi_2_Fe_4_O_9_	0.5	50 mL; 15	PS 0.2 mM	100%	46
Fe_3_O_4_/MoS_2_–O/biochar	0.4	50 mL; 10		90.64%	12
Bi_2_S_3_@ZC-cLDHs	1.0	100 mL; 20	PS 0.25 g L^−1^	90.1%	This paper

#### 3.3.2 Influence of PS dose

The loading of PS influences the degradation of pollutants because to its association with the generation of free radicals such as SO_4_˙^−^ and ˙OH.^50^ In this experiment, the PS load varied between 0.1–0.5 g L^−1^ (Fig. 5D). The photocatalytic efficiency and the LF decomposition rate constantly significantly increased when the PS loading rose from 0.1 to 0.25 mg L^−1^. At a PS loading of 0.1 g L^−1^, the photocatalytic efficiency was 68.9% (*k* = 0.014 min^−1^). At PS loading of 0.25 g L^−1^, efficiency rose to 90.1% and, efficiency slightly improved to 91.1% (*k* = 0.0291 min^−1^) with PS loading of 0.3 g L^−1^. According to eqn (i), increasing the PS loading will result in more S_2_O_8_^−^ interacting with electrons. These generate more ˙OH radicals, hence enhancing the efficacy of LF degradation. Nonetheless, the amount of photogenerated species remains constant for a fixed loading of Bi_2_S_3_@ZC-cLDHs and specific photon output from light; therefore, augmenting the PS loading to 0.3 g L^−1^ may lead to an excess of S_2_O_8_^−^ which cannot function as an electron acceptor in the photocatalytic process, resulting in the photodegradation efficiency attaining a stable level or exhibiting minimal increase.^51,52^

#### 3.3.3 Influence of Bi_2_S_3_@ZC-cLDHs dosage

Fig. 5C illustrates the LF degradation capability of Bi_2_S_3_@ZC-cLDHs with loading ranging from 0.5 to 2.0 g L^−1^. Upon augmenting the Bi_2_S_3_@ZC-cLDHs loading from 0.5 to 1.0 g L^−1^, the degradation efficiency of LF escalated significantly from 59.9% (*k* = 0.0118 min^−1^) to 90.1%. However, further increasing the Bi_2_S_3_@ZC-cLDHs loading to 1.5–2 g L^−1^ resulted in a marginal increase in efficiency at 1.5 g L^−1^ (*k* = 0.0306 min^−1^), followed by a decrease to 82.3% (*k* = 0.0209 min^−1^) at 2 g L^−1^. An increased photocatalytic dose results in a greater generation of electron–hole pairs and a higher production of SO_4_˙^−^ by electron excitation, hence enhancing decomposition efficiency up to a certain threshold. Beyond this point, additional increases in photocatalytic dose lead to a decline in decomposition efficiency. The cause may be attributed to two factors:^51,53^ (i) the quantity of PS supplied is fixed, and by extension, the degradation efficiency is constrained because to the finite amount of SO_4_˙^−^ created by electron excitation. (ii) The catalyst particles clash, obstructing the active sites of other particles and hence restricting the active sites accessible to the LF molecule. Moreover, Furthermore, the range of photons generated from visible light sources is limited when in contact with the suspension.^1,54^

#### 3.3.4 Influence of LF initial concentration

As shown in Fig. 5E, the degradation efficiency was assessed at LF concentrations between 10 and 40 mg L^−1^ with Bi_2_S_3_@ZC-cLDHs loading of 1.0 g L^−1^ and PS loading of 0.25 g L^−1^ for 90 minutes of sunlight. At concentration of 10 ppm, the highest degradation efficiency was 93.3% (*k* = 0.0342 min^−1^), whereas at concentration of 40 ppm, the lowest degradation efficiency was 57.5% (*k* = 0.0114 min^−1^). At concentrations of 20 ppm and 30 ppm, the degradation efficiencies were 90.1% and 78.9% (*k* = 0.0202 min^−1^), respectively. Consequently, establishing a LF concentration of 30 ppm was the optimal setting for the study. The findings indicated that the effectiveness of LF degradation decreased with an increase in LF concentration. For PS and Bi_2_S_3_@ZC-cLDHs, the loading parameters remained constant. The Bi_2_S_3_@ZC-cLDHs photocatalytic system produced almost identical quantities of photogenerated electron–hole pairs as the LF concentration increased from 10 to 40 pm. At low concentrations of LF, ROS rapidly oxidized LF, resulting in enhanced breakdown efficiency. Conversely, when the concentration of PL increased the ROS were more inadequate to deconstruct LF, resulting in a reduced efficiency of LF degradation.^37,53^

### 3.4 Cycling test

**Fig. 6:**
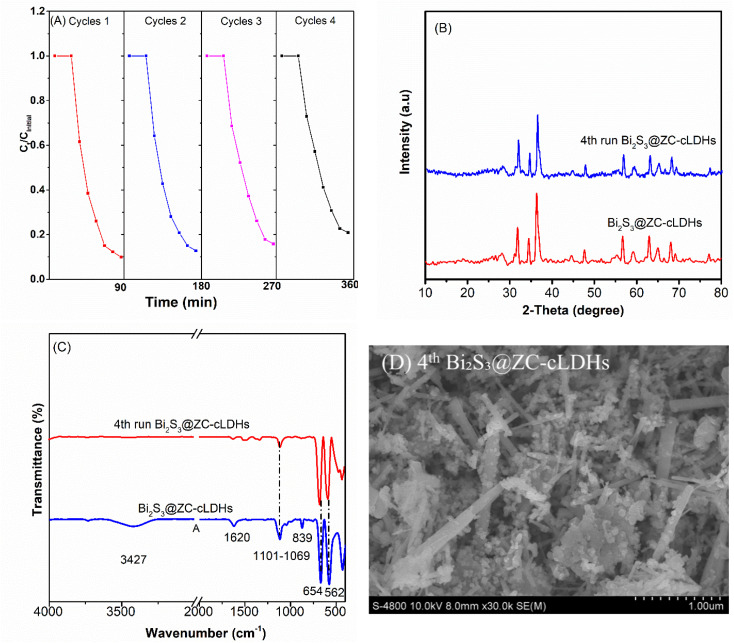
(A) The photocatalytic stability, (B) XRD, (C) FTIR and (D) SEM pattern before and after the 4th recycling of Bi_2_S_3_@ZC-cLDHs.

The durability and reusability of photocatalytic material are essential considerations to put the material into practical applications. Fig. 6A presents the assessment outcomes of the durability and LF degradation efficacy of the Bi_2_S_3_@ZC-cLDHs sample after four reutilizations. The Bi_2_S_3_@ZC-cLDHs, after the first photocatalytic process, was filtered, rinsed with distilled water, dried, and then utilized in the subsequent photocatalytic process under identical catalytic conditions as the first instance. The experimental findings indicate that the photocatalytic effectiveness of Bi_2_S_3_@ZC-cLDHs in degrading LF after 4th reuses decreased in the following sequence: 90.1%, 87.2%, 84.16%, and 79.1%. The XRD and IR spectra (Fig. 6B and C) of the synthesised Bi_2_S_3_@ZC-cLDHs sample before and after the fourth reuse indicate a stable structure, with little changes in the main peaks and a slight decrease in peak strength. The buildup of by-products on the photocatalyst's surface may be the cause; the repetitive washing of Bi_2_S_3_@ZC-cLDHs diminishes the bonding characteristics of Bi_2_S_3_-ZC-cLDHs. SEM image (Fig. 6D)results indicate that upon reuse, the surface area of Bi_2_S_3_@ZC-cLDHs exhibits minor alterations; the Bi_2_S_3_ rods begin to deform, and ZC-cLDHs display agglomeration and uneven dispersion on the rod surface, resulting in modified surface area and consequently diminishing the capacity to interact with light photons.

### 3.5 Photocatalytic mechanism and degradation pathways

#### 3.5.1 Photocatalytic mechanism

Several quenching experiments were trapped to investigate the ROS engaged in the LF degradation of Bi_2_S_3_@ZC-cLDHs. With LF concentration of 20 ppm, PS loading of 0.25 g L^−1^, Bi_2_S_3_@ZC-cLDHs loading of 1.0 g L^−1^, and light irradiation time of 90 minutes, the LF degradation efficiency of PS-activated Bi_2_S_3_@ZC-cLDHs reached 90.1%.

**Fig. 7:**
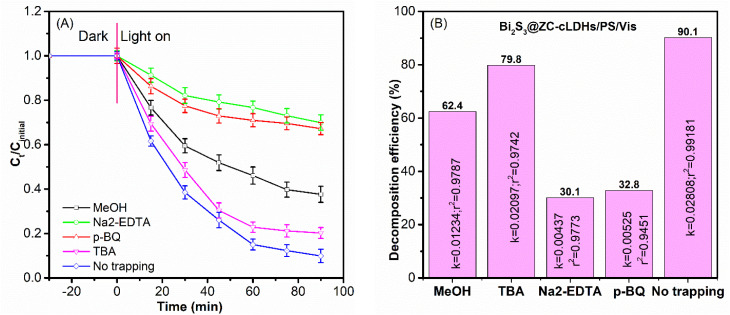
(A and B) Trapping experiments of ROS (h^+^, SO_4_˙^−^ and ˙OH, ˙OH and O_2_˙^−^ using *p*-BQ, MeOH, TBA and Na_2_-EDTA).

In the optimization experiment above, *p*-BQ, MeOH, TBA and Na_2_-EDTA were used as trapping agents to capture O_2_˙^−^; SO_4_˙^−^ and ˙OH, ˙OH; h^+^ respectively, to identify the ROS that are major to the LF degradation process in the Bi_2_S_3_@ZC-cLDHs heterojunction.^11,14^ As shown in Fig. 7A and B trapping agents' incorporation reduced the degradation efficiency of LF. The LF decomposition efficiency dropped little to 62.4% (*k* = 0.01234 min^−1^) when SO_4_˙^−^ and ˙OH were captured with MeOH, and by 79.8% (*k* = 0.02097 min^−1^) when ˙OH was captured with TBA. The degradation rate FL of Bi_2_S_3_@ZC-cLDHs in the presence of MeOH was inferior to that seen with TBA, suggesting that the presence of SO_4_˙^−^ in ROS may augment the degradation FL for Bi_2_S_3_@ZC-cLDHs. However, with *p*-BQ and Na_2_-EDTA, the degradation efficiency decreased significantly to 32.8% (*k* = 0.00525 min^−1^) and 30.1% (*k* = 0.00437 min^−1^). The findings indicated that h^+^ and O_2_˙^−^ were the primary agents in the LF breakdown process. The free radicals breakdown FL in the following order: h^+^ > O_2_˙^−^ > ˙OH > SO_4_˙^−^.

XRD and UV-Vis diffraction analyses revealed that ZC-cLDHs had two phases, ZnO and ZnCo_2_O_4_, with band gap energies measured at 2.97 eV and 2.2 eV, respectively. The conduction band (CB) and valence band (VB) potentials of these two semiconductors were computed using the following eqn (iii) and (iv).^16,55^

iii *E*_VB_ = *X* − *E*_e_ + 0.5 *E*_g_

iv *E*_CB_ = *E*_VB_ − *E*_g_

*X* represents the electronegativity of the catalysts, and *E*_g_ shows the energy of free electrons on the standard hydrogen scale (approximately 4.5 eV).^55^ This results in a VB potential of 2.775 eV and CB potential of −0.195 eV for ZnO; −0.565 eV and 1.445 eV for ZnCo_2_O_4_; and 0.3 eV and 1.82 eV for Bi_2_S_3_. Considering the optical characteristics, calculated band potentials locations of Bi_2_S_3_ and cLDHs, together with the trapping experiments of active species, a plausible mechanism for the photocatalytic activity could be described as follows:

Upon exposure to sunlight, both Bi_2_S_3_ and ZC-cLDHs semiconductors within the Bi_2_S_3_@ZC-cLDHs heterojunction absorb light, resulting in the excitation of electrons in the VB, therefore generating (e^−^/h^+^). In the ZC-cLDHs semiconductor, the CB potential of ZnCo_2_O_4_ is lower than that of ZnO, and both potentials are more negative than the O_2_/O_2_˙^−^ potential 
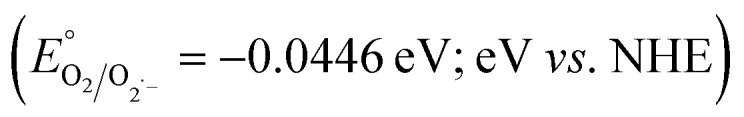
.^30^ Consequently, this excited electrons in the CB of ZnCo_2_O_4_ transfer to the CB of ZnO, where they combine with (e^−^) from ZnO to react with surface-adsorbed O_2_, resulting in the formation of O_2_˙^−^, which subsequently oxidizes LF resulting in products. Furthermore, these charged electrons can also disintegrate straightaway into LF. Moreover, the LF decomposition rate is enhanced, diminishing the recombination of photogenerated electrons and holes, since the S_2_O_8_^2−^ activator can trap this electron produced on the catalyst's surface and produce SO_4_˙^−^ and ˙OH to further decompose LF.

**Fig. 8:**
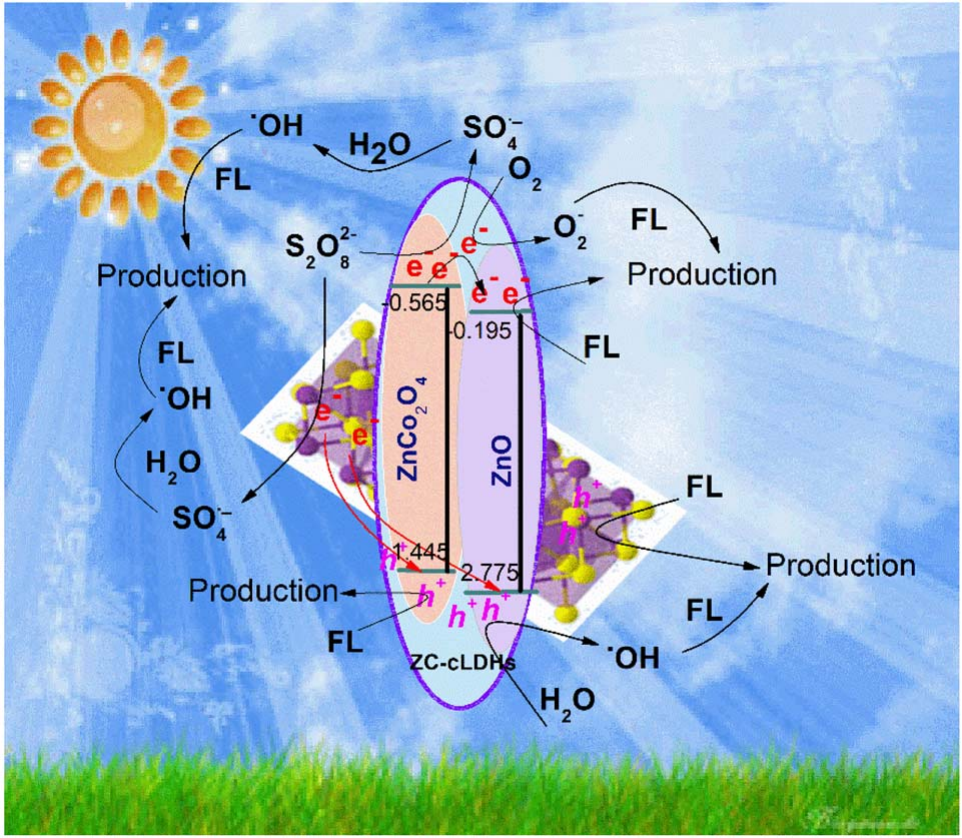
Z-scheme photocatalytic mechanism for Bi_2_S_3_@ZC-cLDHs heterojunction photocatalysts.

For Bi_2_S_3_, *E*_vb_ = 0.3 eV is more positive than the standard potential of O_2_/O_2_˙^−^, but *E*_vb_ = 1.99 eV is lower than the standard potential of ˙OH/H_2_O (*E* = 1.99 eV *vs.* NHE). The (e^−^) are incapable of reacting with O_2_ to produce O_2_˙^−^, and the (h^+^) in the valence band cannot oxidize H_2_O to create. Consequently, the charges produced by light do not traverse the traditional type-II heterojunction charge transfer mechanism. Consequently, they stay trapped inside the Bi_2_S_3_ structure, causing decreased efficiency in the capture and use of solar energy for chemical processes. This is accomplished *via* the direct charge transfer mechanism in alignment with the Z-scheme (Fig. 8). The (e^−^) at the conduction band of Bi_2_S_3_ transition to the VB of ZC-cLDHs, where they combine with the holes of ZC-cLDHs. in addtion, the (e^−^) in the CB of Bi_2_S_3_ may react with S_2_O_8_^2−^ to generate SO_4_˙^−^ and ˙OH radicals, facilitating the degradation of LF. For the ZC-cLDHs, ZnO's *E*_cb_ = 2.775 eV is greater than the standard oxidation potential of ˙OH/H_2_O, which means it react with H_2_O to produce ˙OH to decompose LF. In contrast, ZnCo_2_O_4_'s *E*_vb_ = 1.445 eV is smaller than the standard oxidation potential of ˙OH/H_2_O, meaning it cannot oxidize H_2_O to form ˙OH; instead, the (h^+^) can directly decompose LF to the product.

#### 3.5.2 Degradation pathways

**Fig. 9:**
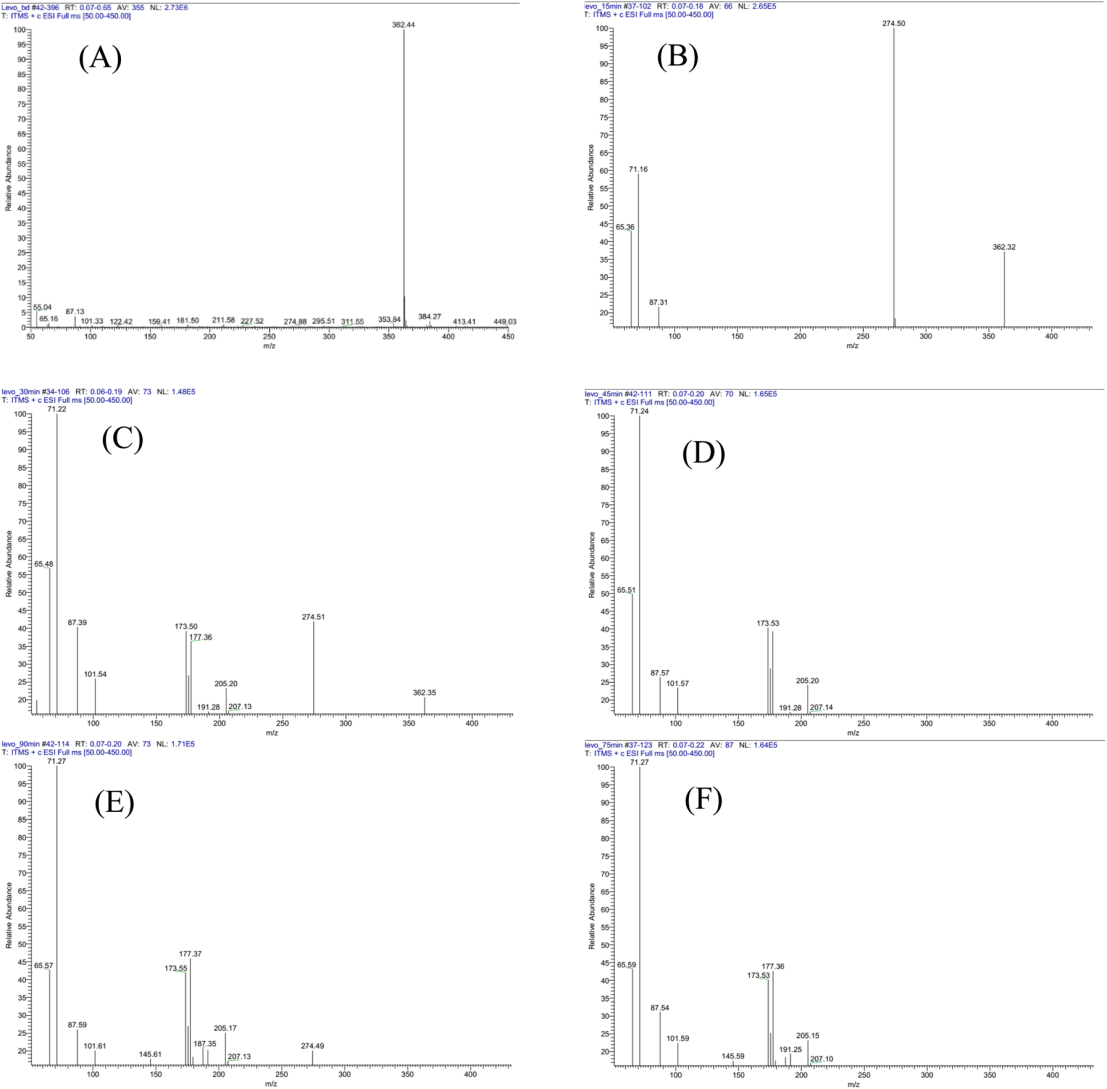
The degradation of LF has performed based on times: (A). *t* = 0 min; (B) *t* = 30 min; (C) *t* = 45 min; (D) *t* = 60 min; (E) *t* = 75 min; and (F) *t* = 90 min.

**Scheme 1:**
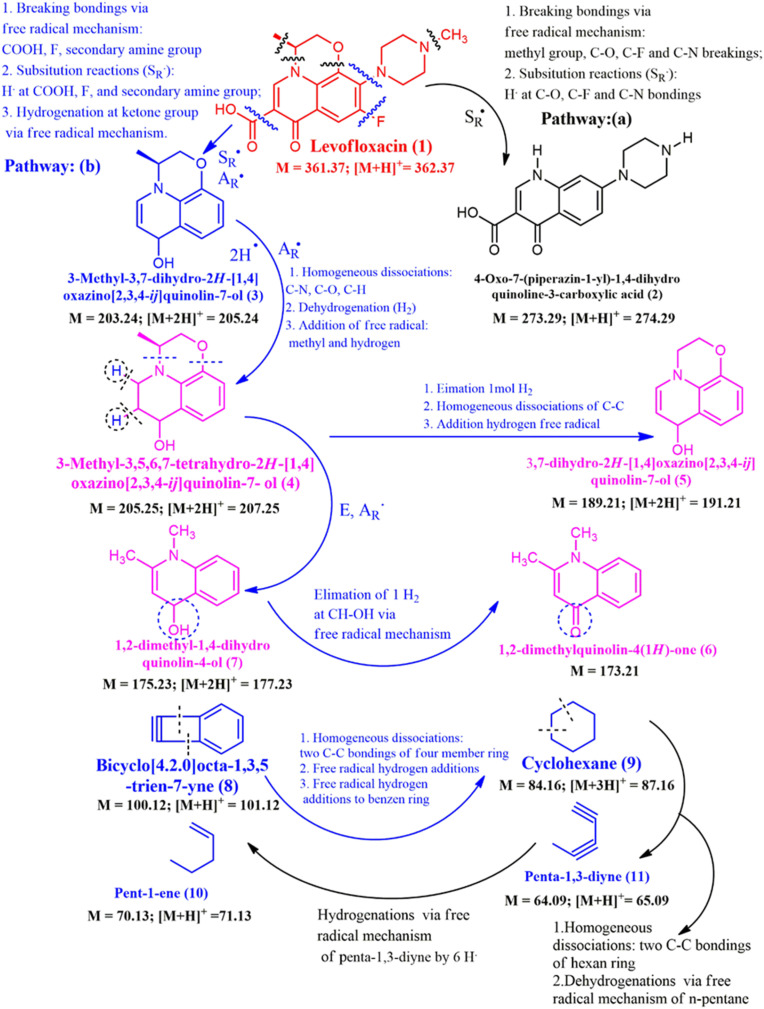
The electron mechanism of LF solution degradations.

The LF degradation has initial concentration of 20 ppm that was conducted in one LTQ XL Linear Ion Trap Mass Spectrometer, UHPLC (Ultimate 3000 MS: LTQ XL), with the positive ionization and ESI-MS. The concentrations of LF are detected at times of *t* = 0, 30, 45, 60, 75, and 90 min as seen in Fig. 9A–F and the results of LF degradation to small molecule ions have been shown in Scheme 1. As seen in Fig. 9A, the predicted values of [M + H]^+^ = 362.37 g mol^−1^ and the experiment value of [M + H]^+^ = 362.37 g mol^−1^ that corresponded to molecule mother ion of LF molecule and proved LF has no degradation (100% relative abundance of mother ion). At time of 30 min, LF has started degradations formed to experiment molecule ions such as 274.50, 71.16, 65.36, and 87.31 that belong to [M + H]^+^ = 274.29 or compound (2) as seen in Scheme 1, [M + H]^+^ = 71.13 or compound (10) as indicated in Scheme 1, [M + H]^+^ = 65.09 or ion fragment (11), respectively, so at time of 30 min, the LF makes the degradations to (2), (b) and (11) and it has reduced to 65% of initial concentration (Fig. 9B).

At time of 45 min, as seen in Fig. 9C, compound (1), the LF has reduced around value of 75% that is transform to ion fragments of 274.51, 205.20, 177.36, 173.50, 101.54, 87.39, 71.22, and 65.48 that belong to [M + H]^+^ = 274.29 (1), [M]^+^ = 205.25 (4), [M+2H]^+^ = 177.23 (7), [M]^+^ = 173.50 (6), [M + H]^+^ = 101.12 (8), [M + H]^+^ = 87.16 (9), [M + H]^+^ = 71.13 (10), and [M + H]^+^ = 65.09 (11), respectively, as seen in Scheme 1. As seen in Fig. 9D–F that are corresponded to times of 60, 75 and 90 min of the degradations and at time of 60 min, the LF almost has been degraded 100%. It has been transferred to small fragment ions such as 205.15, 191.25, 177.36, 173.53 101.59, 87.54, 71.27, and 65.59 that are detected [M + 2H]^+^ (3), c (5), [M + 2H]^+^ (7), [M]^+^ (6), [M + H]^+^ (8), [M + 3H]^+^, [M + H]^+^ (10), and [M + H]^+^ (11), respectively, as seen in Scheme 1. Molecule ions that have lower molecule weights have appeared in higher centrations such as [M + H]^+^ (10), and [M + H]^+^ (11) and has proved that degradations to low molecule weights such as compound (10) and compound (11) are simple alkene and diyne derivatives as seen in Fig. 9D. As seen in Scheme 1, the decompositions of LF or compound (1) to compounds (2–11) has been detected based on LC-MS results at time of 60 min that remained zero percent of LF, *via* reactions that are free radical substitution (S_R_˙), free radical addition (A_R_˙), elimination reaction, and hydrogenation *via* free radical reaction mechanism. The free radicals that have participated in reactions in Scheme 1 are explained *via* active mechanisms in Fig. 7A and B such as ROS.^14,36,56^ All reactions have conducted the Bi_2_S_3_@ZC-cLDHs heterojunction photocatalysts reactions. As exposed in Scheme 1, the reaction of LF (1) has yielded (3) *via* two typical types that have determined the free radical substitution (S_R_˙) and the free radical addition (A_R_˙) by breaking bonding *via* the free radical mechanism: COOH, F, and secondary amine group and substitution reactions (S_R_˙): H atom of COOH, F, and secondary amine group, finally, the hydrogenation at ketone group *via* free radical mechanism. Compound (3) is like compound (P9) or compound (L7) in articles.^2,3^ The transform of (1) to compound (2) has performed by substitution reaction (S_R_˙) that also explained as previous article *via* fragment (P28) or (P5)^2,49^*via* the homogeneous dissociations at C–N, C–O, and C–H bonding, the dehydrogenation, and addition of free radial of methyl and hydrogen, compound (3) has transformed to (4) that is similar to degradation as compound (P13) in article.^49^ Compound (4) has yielded compound (7) *via* E and A_R_˙ reactions and it also transformed to (5) *via* elimination of 1 mol H_2_, homogenous dissociation of C–C bonding and additional hydrogen *via* free radical reaction. Fragment (7) to (6) has been detected by the elimination of 1 mol H_2_ at CH–OH bonding *via* free radical mechanism reaction.

The small compound (8) has yielded compound (9) *via* some mechanisms such as homogeneous dissociations of two C–C bonding of four-member rings, free radical hydrogen additions, and free radical addition of hydrogen atoms to benzene derivatives. Compound (9) has the transform to compound (11) by mechanism and compound (11) has transformed to compound (10) *via* some mechanisms in Scheme 1. Compounds (8), (9), (10), and (11) are small compounds and compounds (10) and (11) are expecting compounds of degradation. Our project expects these compounds will be changed to CO_2_ and H_2_O, but the final analysis result of GC-MS has not been detected. The degradation of LF (1) to small molecular weight such as compound (10) to (11) as GC-MS results and proposal mechanism reactions exposed advances in degradation mechanism of LF that compared to the former articles.^1,3–5^

## 4 Conclusion

The synthesis of the novel Bi_2_S_3_@ZC-cLDH heterojunction was accomplished effectively. ZC-cLDHs include two phases, ZnO and ZnCo_2_O_4_, uniformly distributed on the surface of Bi_2_S_3_ rods. The photoactivity of the Bi_2_S_3_@ZC-cLDHs heterojunction was greatly enhanced by the addition of PS; the combined action of Bi_2_S_3_@ZC-cLDHs and PS activated PS to function as an electron acceptor, facilitating the separation of photogenerated electron–hole pairs. As a result, the LF decomposition rate of Bi_2_S_3_@ZC-cLDHs/PS/Vis increased by 2.2 times the rate of Bi_2_S_3_@ZC-cLDHs/Vis. The combination of Bi_2_S_3_ and ZC-cLDHs significantly improved the degradation of LF under visible light, shown by the LF degradation rate of the Bi_2_S_3_@ZC-cLDHs/PS/Vis system being 1.6 times superior to that of the ZC-cLDHs/PS/Vis system. High charge separation and transfer efficiency have been predicted by the Z-scheme for the LF degrading process of Bi_2_S_3_@ZC-cLDHs/PS/Vis. The Bi_2_S_3_@ZC-cLDHs demonstrated significant stability after four reuses and exceptional reusability.The degradation products resulting from the LF photodegradation of Bi_2_S_3_@ZC-cLDHs were identified as uncomplicated and low-toxicity compounds. The Bi_2_S_3_@ZC-cLDHs heterojunction with PS activator under visible light is shown to be an effective and environmentally friendly method for removing LF from water in this study.

## Author contributions

Nguyen Thi Mai Thoparticipated in methodology, validation, writing, reviewing, and project administration. Minh An Tran Nguyen participated in editing the draft manuscript.

## Conflicts of interest

The authors claim that they have no known competing interests that could have an impact on the research described in this publication.

## Data Availability

We send the DAS of the article “Manuscript ID: RA-ART-05-2025-003086 TITLE: construction of novel Bi_2_S_3_@Zn-Co-cLDHs heterojunction for enhanced photocatalytic degradation of levofloxacin with persulfate activation under visible light: mechanism, degradation pathway” https://doi.org/10.6084/m9.figshare.28916051.
